# A comprehensive promoter landscape identifies a novel promoter for CD133 in restricted tissues, cancers, and stem cells

**DOI:** 10.3389/fgene.2013.00209

**Published:** 2013-10-29

**Authors:** Ramakrishna Sompallae, Oliver Hofmann, Christopher A. Maher, Craig Gedye, Andreas Behren, Morana Vitezic, Carsten O. Daub, Sylvie Devalle, Otavia L. Caballero, Piero Carninci, Yoshihide Hayashizaki, Elizabeth R. Lawlor, Jonathan Cebon, Winston Hide

**Affiliations:** ^1^Department of Biostatistics, Harvard School of Public HealthBoston, MA, USA; ^2^Harvard Stem Cell Institute, Faculty of Arts and Sciences, Holyoke CenterCambridge, MA, USA; ^3^The Genome Institute, Washington University School of MedicineSt. Louis, MO, USA; ^4^Ludwig Institute for Cancer ResearchHeidelberg, VIC, Australia; ^5^RIKEN Omics Science Center, RIKEN Yokohama InstituteKanagawa, Japan; ^6^Department of Cell and Molecular Biology, Karolinska InstituteStockholm, Sweden; ^7^RIKEN Center for Life Science Technologies, Division of Genomic TechnologiesYokohama, Kanagawa, Japan; ^8^Ludwig Institute for Cancer Research Ltd., New York Branch at Memorial Sloan-Kettering Cancer CenterNew York, NY, USA; ^9^Ludwig Collaborative Laboratory, Department of Neurosurgery, Johns Hopkins UniversityBaltimore, MD, USA; ^10^RIKEN Preventive Medicine and Diagnosis Innovation ProgramWako, Saitama, Japan; ^11^Departments of Pediatrics and Pathology, University of MichiganAnn Arbor, MI, USA

**Keywords:** PROM1 protein, human, AC133 antigen, transcription start site, promoter regions, genetic, melanoma, cancer stem cells

## Abstract

PROM1 is the gene encoding prominin-1 or CD133, an important cell surface marker for the isolation of both normal and cancer stem cells. PROM1 transcripts initiate at a range of transcription start sites (TSS) associated with distinct tissue and cancer expression profiles. Using high resolution Cap Analysis of Gene Expression (CAGE) sequencing we characterize TSS utilization across a broad range of normal and developmental tissues. We identify a novel proximal promoter (P6) within CD133^+^ melanoma cell lines and stem cells. Additional exon array sampling finds P6 to be active in populations enriched for mesenchyme, neural stem cells and within CD133^+^ enriched Ewing sarcomas. The P6 promoter is enriched with respect to previously characterized PROM1 promoters for a HMGI/Y (HMGA1) family transcription factor binding site motif and exhibits different epigenetic modifications relative to the canonical promoter region of PROM1.

## Introduction

Surface markers play an important role in the purification of stem and progenitor cells. CD133, (PROM1) is a transmembrane protein (Corbeil et al., 2001; Shmelkov et al., 2005) that is widely used as a cell-surface marker for stem cell and cancer stem cell populations (Bussolati et al., 2005; Lee et al., 2005; Tirino et al., 2008; Zhang et al., 2008). Originally identified in hematopoetic progenitor cells by the AC133 antibody (Miraglia et al., 1997; Yin et al., 1997; Fargeas et al., 2003) CD133 displays restricted expression in both adult human (Shmelkov et al., 2004; Florek et al., 2005) and adult mouse tissues (Mizrak et al., 2007). CD133^+^ cells have also been broadly identified in non-hematopoetic tissues during differentiation *in vitro* and *in vivo* (Bussolati et al., 2005; Lee et al., 2005; Snippert et al., 2009) and have been isolated from brain and other cancers that possess stem-cell properties. For some tumor types (e.g., brain, liver, and Ewing sarcoma) CD133^+^ cell populations have been reported to be more tumorigenic than CD133^−^ cell populations in xenograft assays (Singh et al., 2004; Jiang et al., 2010; Tang et al., 2011; von Levetzow et al., 2011). However, in colon cancers CD133^+^ and CD133^−^ populations have been found to be equally capable of tumor initiation in xenografts (Shmelkov et al., 2008), and both cell fractions have substantial tumor initiating activity in melanoma, lung, and ovarian cancer (Meng et al., 2009; Shackleton, 2010; Stewart et al., 2011), making CD133 a controversial marker for cancer stem cells (LaBarge and Bissell, 2008). Recently, a functional role for CD133 in suppression of neuroblastoma differentiation has been described (Takenobu et al., 2010), further complicating the understanding of its role and value as a suitable surface marker.

**Figure 1:**
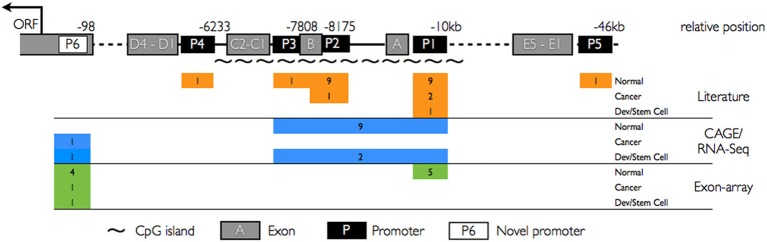
**Summary of PROM1 promoter architecture and cross-platform activity**. Upper panel: CD133 promoter structure with known promoter regions P1–P5 (black rectangles), novel proximal promoter P6 (white rectangle), known exons A–E (gray rectangles), and CpG islands (~). Lower panel: Promoter activity (colored rectangles, number of studies expressing that category) in normal tissues, cancer, and developmental systems/stem cells as captured by different platforms. Orange: known promoter activities reported in the literature based on single gene studies. Blue: CAGE and RNA seq studies. Green: Affymetrix exon array exome expression. Promoters P1–P3 are widely expressed in the literature and using CAGE and RNA-seq assay. P6 is not yet reported in the literature.

To date, using sampling across limited types of tissues and cancers, five TATA-less promoters (P1–P5) have been identified in the 5′ upstream region of PROM1. These promoters differentially regulate expression of PROM1 in adult tissues and cancer cell types (Shmelkov et al., 2004). The most distally located promoter, P5, is present at 46 kb from the start codon followed by P1, P2, P3, and P4 promoters which are present at a distance of −10, −8, −7.8, and −6 kb, respectively (Figure 1). Promoters P1 and P2 drive PROM1 transcription in kidney, liver, pancreas, placenta, lung, spleen, and colon, but can also exhibit tissue restriction with P1 activity in small intestine and prostate whereas P2 is active in brain and ovary (Table 1); P3 is rarely active and has only been reported in skeletal muscle, P4 and P5 activity appears to be restricted to testis (Shmelkov et al., 2004). Further characterization has shown that P1, P2, and P3 promoters contain stretches of CpG islands under epigenetic regulation (Pleshkan et al., 2008; Tabu et al., 2008; Pellacani et al., 2011) under transcriptional control of Sp1 and Myc (Gopisetty et al., 2012). Collectively these findings suggest that PROM1 expression is tightly regulated in adult tissues through the choice of specific promoters across different cell types. However, additional relationships between choice of promoter, regulatory elements, and expression restriction in normal and malignant tissues have yet to be determined.

**Table 1:** **PROM1 alternative promoters and regulation**.

**Promoter**	**Genomic location**	**Distance from start codon^c^**	**Tissue**	**Transcription factors**	**References**
P1^a^, ^b^	16085637 – 16087537	−10008	Fetal liver, liver, kidney, pancreas, placenta, lung, spleen, colon, small intestine, and prostate Glioblastoma, lung cancer	OCT4, SOX2 (during hypoxia), Sp1/Myc	Shmelkov et al., 2004; Iida et al., 2011; Gopisetty et al., 2012
P2^a^, ^b^	16085338 – 16085704	−8175	Brain, ovary, kidney, liver, pancreas, placenta, lung, spleen and colon Glioblastoma	Sp1/Myc	Shmelkov et al., 2004; Tabu et al., 2008; Gopisetty et al., 2012
P3^a^	16084913 – 16085337	−7808	Skeletal muscle	Sp1/Myc	Shmelkov et al., 2004; Gopisetty et al., 2012
P4	16082163 – 16083762	−6233	Testis	Not studied	Shmelkov et al., 2004; Tabu et al., 2008
P5	16122394 – 16123893	−46364	Testis	ETS	Shmelkov et al., 2004; Tabu et al., 2008, 2010
P6	16077254 – 16077627	−98	Cancer, stem cells, and retina	HMG	–

a Contains CpG island.

b Hypomethylated in glioblastomas.

c Start codon at location 16077529 in Exon 2 (16077741 – 16077309).

As cancer cells can acquire the properties of stem cells, and contain a stem-like population marked with CD133, a comprehensive understanding of the differential utilization of PROM1 promoters that regulate the expression of CD133 may illustrate the differences in its expression within populations of cells with stem-like phenotypes. In order to more broadly determine aspects of PROM1 regulation and to identify key regulatory elements associated with PROM1 expression in cancer and stem cells, we have used Cap Analysis of Gene Expression [CAGE, (Kodzius et al., 2006)] to perform an exhaustive assessment of the landscape of the PROM1 upstream promoter region. CAGE precisely defines the location of transcription start sites (TSS) by sequencing from the 5′ end of capped, full-length mRNA. In addition to TSS identification, CAGE can measure transcript abundance, allowing comparison of promoter activity between samples.

To gain additional insight into PROM1 promoter activity in the context of cancer cells with stem-like properties we have performed high-coverage CAGE sequencing of five melanoma cell lines directly derived from patient biopsies (Table S2), sorted by CD133^+^ into a small minority of cells from the total CD133^−^ population (Gedye et al., 2009). The promoter activity in these populations was compared with a panel of CAGE libraries derived from 72 tissues and cell types, including 13 normal tissues, 25 cancer tissues, and 34 developmental states (Table S1).

We identify a previously unknown promoter that shows differential expression and regulation of PROM1 mRNA in restricted tissues, stem-like cells within cancer cell lines and stem cells.

## Materials and methods

### Cell culture

To isolate melanoma cells from fresh human melanoma explants, freshly excised human melanoma specimens were inspected by pathologists and fragments removed for cell line establishment without disturbing surgical margins. The melanoma cell lines were derived from metastatic melanoma tissue and used before passage 10. Description of the cell lines and associated gene-expression data have been reported previously (Behren et al., 2013). Patient consent was collected and ethical approval for the use of the cell lines has been granted by the Austin Health Human Research Ethics Committee (HREC). Cell lines were cultured in our standard media (“RF10”) comprising RPMI 1640 supplemented with 2 mM Glutamax®, 25 mM HEPES, 50 μM 2-mercaptoethanol (Hamburger and Salmon, 1977), 100 U/mL penicillin, 100 μg/mL streptomycin (all from Invitrogen, Mulgrave, Australia) plus 10% fetal calf serum (FCS; from CSL, Melbourne, Australia). Tissue fragments were mechanically dissociated and passed through a cell strainer, remaining fragments were subjected to enzymatic digestion in a collagenase/DNAse/serum-free digestion media mixture overnight at 37°C and single cell suspension plated out the next morning. Once established the cell lines were HLA-typed by the Red Cross in Melbourne to ensure the match with donor tissue and were tested for *mycoplasma* contamination. Harvested cells were washed, counted and plated into 96 well round bottom plates at 10^4^–10^5^ per well. After pelleting by centrifuge the cells were washed once with PBS cells and blocked in 50 μ L PBS/10% normal human serum for 10 min. The plate was gently vortexed to resuspend cells and 1 μ L of AC133-PE antibody (Miltenyi Biotec, Bergisch Gladbach, Germany) was added to each well prior to incubation at 4°C for 15 min. Cells were washed and resuspended and immediately analyzed on a FACSCalibur flow cytometer (Becton Dickson, San Jose, CA). An anti-CD4-PE antibody was used at same concentration as Isotype control. The five different melanoma cell cultures derived from biopsy specimens of patients with malignant melanoma were evaluated for CD133 expression by immuno-histochemistry (IHC) and flow cytometry (Table S2).

### Cell separation and RNA extraction

Cell separation was performed by magnetic bead antibody labeled in the cell separation MACS buffer prepared according to the manufacturer's instructions (PBS pH 7.4 with 0.5% BSA and 0.5 mM EDTA). MACS columns were refrigerated for at least 1 h prior to use. Positive selection of cells was performed using LS columns followed by depletion with LD columns. 10^7^ cells were resuspended in 80 μ L MACS buffer; 20 μ L FcR blocking reagent +20 μ l of directly conjugated CD133 beads added, mixed well, and incubated for 30 min at 4°C. Labeled cells were washed, resuspended, and applied to the column. To increase purity columns were run in serial. Cells were passed through LS columns and were then applied to LD columns as “pre-depleted” cells. The cell population selected by the first LS column was then applied to a second LS column to increase enrichment. After separation all fractions were stained as described and purity of subpopulation measured by flow cytometry.

RNA was extracted from 10^7^ purified CD133^+^ or CD133^−^ cells using TriReagent following manufacturer's protocol (Molecular Resarch Center, Inc., Cincinnati, OH, USA). Briefly, cells were homogenized in Trireagent, RNA collected in the aqueous phase after addition of chloroform and precipitated by isopropanol addition. RNA was quality checked by gel electrophoresis and quantified using a nanodrop.

### Gene expression transcript analysis

CAGE was performed as described previously (Kodzius et al., 2006; Kawaji et al., 2009). Total RNA extracted from CD133^+^ and CD133^−^ melanoma cells was used to synthesize the cDNA. RNA and cDNA pools were treated with RNAse I to cleave all ssRNA, leaving only full length cDNA/RNA hybrids for capture with biotin-streptavidin interactions in an cDNA/RNA hybrid enrichment process called as cap-trapping. In this process full length cDNAs are then ligated with specific linker oligos containing MmeI restriction sites and the second strand cDNA is synthesized. Double-stranded cDNAs are digested with MmeI creating ~20nt of cDNA sequence attached to a 5′ linker. After ligation of the second linker XmaJI to MmeI-cleaved 3′ ends of cDNA, fragments are subjected PCR amplification and restriction site digestion to obtain CAGE sequencing tags (see Kodzius et al., 2006, for details). The resulting CAGE tags were then concatenated and cloned into pZEr0-2 plasmids (Invitrogen) for sequencing. Sequence reads were extracted, filtered and aligned to the hg18 genome build using Nexalign (Lassmann, http://genome.gsc.riken.jp/osc/english/dataresource/), following the methods described in (Kawaji et al., 2009). TSS in the upstream PROM1 gene region based on CAGE were identified from clustered sequence reads using HPeak (Qin et al., 2010) and mapped to known PROM1 promoters extracted from GenBank gene records (AY275524, AY438641, AY438640), resulting in the confirmation of known promoters P1–4 and identification of a novel promoter, P6. Start sites identified by HPeak were compared to *in silico* TSS predictions in the oPOSSUM (Ho Sui et al., 2007) and SwitchGear [UCSC Genome Browser track, (Karolchik et al., 2008)] database.

### Rapid amplification of cDNA ends (5′-race)

To confirm the novel PROM1 TSS, 5′-RACE PCR was performed according to the manufacturer's protocol (invitrogen). RNA was prepared from a CD133^+^ LM-Mel-34 melanoma cell line by ligating the RNA with a 5′ RACE adapter 5′-GCU-GAU-GGC-GAU-GAA-UGA-ACA-CUG-CGU-UUG-CUG-GCU-UUG-AUG-AAA-3′); and a single-stranded cDNA was generated. Two CD133 specific anti-sense primers were chosen from exon2 for a nested PCR to enhance specificity and to obtain a sufficient amplification product. Primers were designed using Primer3 software (Rozen and Skaletsky, 2000), and checked for uniqueness by querying against the human genome using BLAST (Altschul et al., 1990) (Table 2). Amplification of 5′-RACE cDNA was performed with nested reverse primers of PROM1 and adapter specific primers with 1 μ l of the first-strand cDNA reaction. Amplified products were separated on an agarose gel and visualized by ethidium bromide staining.

**Table 2:** **Primers used for validating CD133 novel TSS**.

**Location**	**Forward primer (5′-3′) (specific to RACE adapter)**	**Reverse primer (5′-3′) (specific to CD133)**	**Product size**
Outer	GCTGATGGCGATGAATGAACACTG	CAAGCCTTAGGAGCATCTGTGGAT	177 bp
Inner	AACACTGCGTTTGCTGGCTTTGATG	GCTAGCAAGATCCTCCAAACATGA	62 bp

5′ RACE products were cloned into the pcDNA3.1 TA cloning vector and transformed into bacteria. The clones of each transformation were subjected to colony PCR and the sequencing of inserts was carried out with RACE adapter primers and specific reverse primers. Sanger sequence products of RACE PCR amplified fragments were separated and aligned to the human genome using BLAT (Kent, 2002) together with CAGE mapped TSS to confirm unique mapping to the target sequence.

### Exon arrays

Human embryonic stem cell-derived neural crest stem cells (hESC-NCSC), human adult bone marrow-derived mesenchymal stem cells as well as hESC-NCSC differentiated for 6 weeks *in vitro* together with three independent CD133-FACS-sorted cell populations from STA-ET-8.2 Ewing sarcoma cells (Table S3) were profiled by Affymetrix Human Exon 1.0 (HuEx) arrays as previously described (Jiang et al., 2010). HuEx arrays generated from four primary Ewing sarcomas (tumor RNA graciously provided by Tissue Biorepositories at Children's Hospital Los Angeles and the Children's Oncology Group) were also included for analysis. HuEx data for additional adult tissues was obtained from an Affymetrix tissue panel (http://www.affymetrix.com/support/technical/sample_data/exon_array_data.affx). HuEx data was RMA normalized using BioConductor (package affy) and probe intensities for probes covering PROM1 promoter regions P1–P6 were compared to identify differences in TSS utilization.

### Promoter analysis

To characterize regulatory motifs of the novel PROM1 promoter P6; an additional set of proximal promoters (−300/+100 bp) was selected from a total of 149 TSS found to be co-upregulated with P6 in at least four out of five CD133^+^ of the melanoma cell lines. The 149 TSS were selected based upon a significant difference in CAGE peaks in LM-MEL14/34/42/47/62 from five different patients, CD133^+^ over CD133^−^ as determined by HPeak, (*p* ≤ 0.05, Table S4). The TSS set was tested for nucleotide motif enrichment using MEME (Bailey et al., 2010) (motif width 4–21 nucleotides, both strands, any number of repetitions, *p*-value ≤ 0.05) and compared to a random background distribution of 10,000 CAGE-based proximal promoters taken from the FANTOM4 collection (Kawaji et al., 2009). Significant motifs were tested for overlap with the JASPAR 2009 Core Transcription Factor collection (Sandelin et al., 2004) using TomTom (Bailey et al., 2009). Additional experimentally determined transcription factor binding sites from the ENCODE TF ChIP-seq collection (ENCODE Project Consortium, 2004) were retrieved from the UCSC Genome Browser (hg18, update 2010-06-24, track wgEncodeRegTfbsClustered).

### Epigenetic changes and RNA-seq

CpG island information and differential methylation in the Encyclopedia of DNA Elements (ENCODE) project were retrieved from the UCSC Genome Browser's summary track (Ernst and Kellis, 2010).

## Results

### Existing promoter landscape of PROM1 and a novel promoter in melanoma cell lines

**Figure 2:**
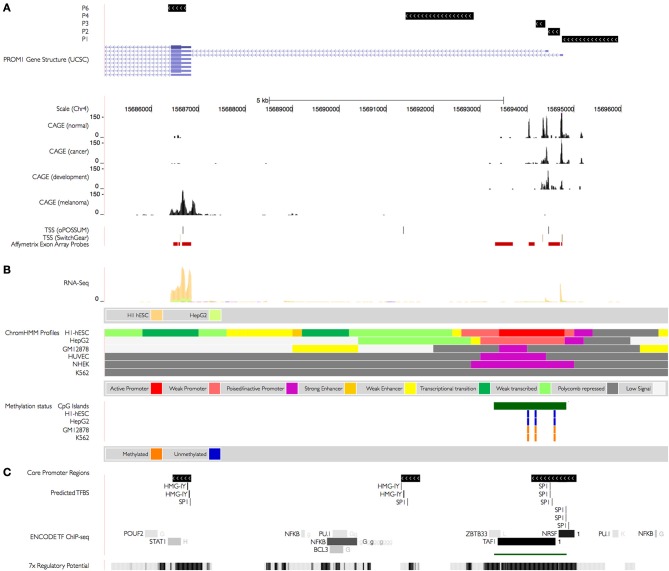
**PROM1 regulatory landscape. (A)** Previously described PROM1 TSS (Promoter 1–3, 4) supported by Capped Analysis of Gene Expression (CAGE) assays from the FANTOM3 collection 57 containing 72 experimental samples of which 13 from normal tissues, 25 from different cancers, and 34 are from developmental states (Table S3). Non-pathogenic tissue CAGE tags are distributed evenly whereas those obtained from tumors are biased toward promoter 1 and 2; transcription in embryonic tissues is biased toward promoter 2. Promoter 6 is supported by CAGE tags obtained from melanoma cell lines and normal colon, small intestine, and rectum. TSS for promoters 1–4 and promoter 6 are supported by predictive models from the SwitchGear and the oPOSSUM database. Exon array probes used to characterize differential exon usage (see Figure 4) are highlighted in red. **(B)** Epigenetic changes in PROM1 promoters. Data from ENCODE tier 1 human cell lines (H1-hESC: embryonic stem cell line, HepG2: liver carcinoma, GM12878: lymphoblastoid, HUVEC: umbilical vein endothelial cells, NHKE: kidney epithelium, K562: myelogenous leukemia). Promoters 1–3 are supported by active (weak) promoters in H1-hESC (HepG2) cells respectively, with stronger expression in H1-hESC as indicated by matching RNA-seq. PROM1 expression from all promoters is polycomb-repressed in GM12878, HUVEC, NHKE, and K562 resulting in no discernible expression. Expression in H1-hESC cells is enhanced at promoter 6, indicating an independent TSS unrelated to transcription driven by canonical promoters P1–3. The known CpG-island (REF) associated with the canonical promoters is unmethylated in both H1-hESC and HepG2 and methylated in GM12878 and K562, further repressing expression in those cells. **(C)** Regulatory potential. A strong regulatory potential is detected around the proximal promoter and promoter 1–3 (ESPERR, REF), with confirmed binding sites for transcription factors POUF2, STATS1, NFKB, and others in the ENCODE tier 1 samples (1: H1-hESC; G: GM12878; g: GM12891/GM12892/GM15510/GM18505/GM18526/GM18951/GM19099/GM19193; H: HeLa-S3; K:K562; L:HepG2). Binding sites for transcription factor found to be enriched in the proximal promoters (+300/−100 nucleotides) of 149 TSS found to be co-activated in CD133^+^-melanoma cells classify the PROM1 promoters into SP1-rich (promoters 1–3, in agreement with the CpG-island) or HMG-IY-rich (promoter 6, promoter 4).

To determine PROM1 promoter utilization across diverse tissues at high resolution we analyzed CAGE tags obtained from CD133^+^ melanoma cell lines and an additional 72 samples grouped as cancer, normal adult tissues and developmental stages from the public FANTOM4 data set (Table S1). Our CAGE analysis confirmed known promoters and identified a novel, sixth promoter (P6) close to the translational start codon (AUG), strongly upregulated in CD133^+^ melanoma cells and with weak expression in normal colon and small intestine libraries (Figure 2A). We determined that TSS marked by CAGE tags from cancer, normal adult tissues, and developmental stages were consistent with previous reports showing P1–P3 to be widely expressed canonical promoters. CAGE tags revealed utilization of all three promoters in normal tissues, whereas promoter utilization cancer samples are biased toward P1/P2, and developing tissues are biased toward P2. These promoter profiles are consistent with previous reports (Table 1). The CAGE tissue panel includes one low coverage testis library with insufficient CAGE tags to support a previously reported testis-specific P4 TSS (Shmelkov et al., 2004).

### Validation of novel promoter

**Figure 3:**
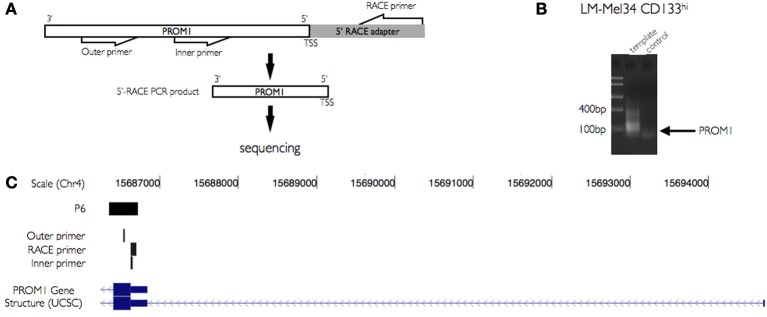
**Validation of novel PROM1 TSS using 5′ RACE PCR. (A)** Schema illustrating primer design for PROM1 TSS validation. PROM1 TSS is amplified using a pair of outer and inner primer sets targeted specifically to 5′ Rapid amplification of cDNA ends (RACE) adapter and PROM1 sequences. **(B)** The 5′ end regions of PROM1 mRNA were amplified from CD133^+^ and CD133^−^ populations of LM-Mel-34 cell lines. Gel electrophoresis of amplified products shows difference in expression level in CD133^+^ and CD133^−^ populations. Amplified 5′ends of PROM1 RNA were cloned in to plasmid pcDNA3.1 and then sequenced. **(C)** UCSC genome browser view of sequenced 5′ end region of PROM1 mapped to the promoter. The tracks shown here illustrates CAGE captured TSS regions from CD133^+^ melanoma cell lines and the RACE PCR identified 5′ end of the PROM1 transcript. The other tracks show outer and inner RACE PCR primers specific to PROM1.

To confirm the initiation of transcripts at P6 in CD133^+^ sorted cells from the melanoma cell lines we used 5′-RACE PCR. mRNA isolated from CD133^+^ cells derived from the melanoma cell line LM-MEL-34 was used to amplify the 5′ end of the PROM1 transcript with the help of a pair of forward primers targeted to a RACE adapter and the other pair of reverse primers specifically targeted to 5′ exon of PROM1 (Table 2, Figure 3A). Gel electrophoresis of amplified products from these cells showed a stronger band with the expected size of 62 bp (Figure 3B). There was no sign of non-specific product in the negative control. Further, the amplified product was cloned into pcDNA3.1 plasmid for sequencing. The sequenced insert region was then mapped to the PROM1 promoter facilitating the TSS identification and the approximate location of promoter elements (Figure 3C). 5′ RACE PCR efficiently detected the initiation RNA transcripts at P6.

### Cross platform evidence for a novel P6 promoter

**Figure 4:**
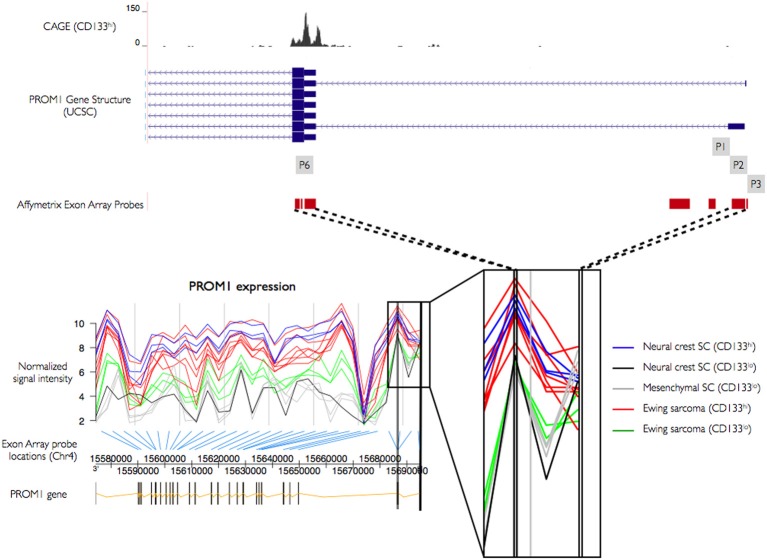
**PROM1 promoter activity using exon arrays**. Transcript wide expression pattern of PROM1 measured by Affymetrix exon arrays with specific probes targeting exonic regions. Y-axis: normalized signal intensity (expression level). X-axis: genomic coordinates of the transcript. Expression of 5′ probes in CD133^+^ neural crest stem cells (blue), Ewing sarcoma (red), and CD133^−^ neural crest stem cells (black), mesenchymal stem cells (gray), and Ewing sarcoma (green). Thick black bars: Probe regions overlapping CAGE defined TSS.

The novel P6 promoter is supported by *in silico* TSS predictions from the oPOSSUM and SwitchGear databases. In order to further explore its utilization we studied human exon array expression data from mesenchymal stem cells (bone marrow MSC), neural crest stem cells (undifferentiated NCSC), Ewing sarcoma (primary tumors and sorted cells, STA-ET-8.2), and an Affymetrix panel of 12 adult tissues. Affymetrix Human Exon 1.0 (HuEx) arrays have an average of four probes per known exon, and seven probes cover the 5′ region of PROM1, four of which showed significant expression, one matching the P1–P2 location and three cover the novel P6 promoter region. Using expression information from these probes we classified promoter utilization across different cell types, testing for stronger expression (higher probe intensity) at P6 than at upstream promoters P1–P2. We found increased expression of probes at the P6 promoter compared to P1–P2 in CD133^+^ Ewing sarcoma cells, mesenchymal stem cells, and undifferentiated NCSC, all of which express high levels of CD133. In contrast, CD133^−^ sorted Ewing sarcoma cells, CD133^−^ mesenchymal stem cells, and differentiated neural crest stem cells show no significant difference in intensities between probes covering P1–P2 and P6 (Figure 4). We found higher expression at P6 in four tissues (colon, pancreas, kidney, and testis), with inconsistent replicate patterns or no discernible difference between P1–P2 and P6 in the other tissues (Figure S1).

## Novel promoter regulation

### Transcription factor binding site (TFBS) motif enrichment

We performed an enrichment analysis of proximal promoters from 149 TSS in genes in the CD133 enriched CD133^+^ melanoma cell lines which were found to be co-upregulated with P6 in at least four of five CD133^+^ cell lines (see methods). We identified three significant motifs characterized as binding sites for AZF/HMG-I/Y, Sp1, and Klf4, two of which (AZF/HMG-I/Y and Sp1) are present in the P6 core promoter region (Figure 2C) and are evolutionarily conserved between human, chimp, and mouse.

### Epigenetic modifications of PROM1 promoter observed in ENCODE data

Epigenetic modifications are key factors for regulation of gene transcription. Since HMG-I/Y has a role in regulating chromatin structure we explored epigenetic modifications of the PROM1 promoter landscape in the publicly available ENCODE consortium dataset of genome signals. Hypermethylation of CpG islands close to P1–3 has been linked to CD133 expression in glioma stem cells and cell lines (Gopisetty et al., 2012). Differential methylation of the same region observed in ENCODE cell lines indicate an unmethylated CpG island in ENCODE cell lines H1-hESC (embryonic) and HepG2 (liver) and methylation of the same CpG island in K562 (blood, leukemic) and GM12878 (blood, lymphoblastoid), in agreement with their RNA-seq expression status in the same ENCODE cell lines (Figure 2B). The novel P6 promoter region does not overlap known CpG islands; in particular, RNA-seq data indicates independent transcription from P6 in H1-hESC. We explored histone modifications as an alternative regulatory mechanism using summary information generated by ChromHMM (Figure 2B, Figure S1), an algorithm that characterizes chromatin states by integrating multiple ChIP-seq histone modification data sets (Ernst et al., 2011). Observed histone changes support an enhancer upstream of P6 as well as transcriptional activity around P1–3 and P6, whereas P1–3 appears poised or repressed in all other ENCODE cell lines represented, mostly due to H3K27me3 and H3K36me3 silencing. In summary, an ensemble of histone methylation marks, RNA pol II binding sites and sequence conservation observed in the PROM1 promoter region support the likelihood of transcript initiation at the P6 promoter.

## Discussion

Although CD133 is widely used as a stem cell marker, its significance and relationship to cancer cells with stem-like properties is controversial (Wu and Wu, 2009; Campos et al., 2011). Previous studies have established five alternative promoters (P1–P5) which drive CD133 expression in normal tissues and cancer cell lines. This transcriptional complexity raises questions in relation to differential regulation of the alternative promoters, a genetic feature which has recently been widely reported (Davuluri, 2008; Pal et al., 2011). To provide a coherent overview of PROM1 promoter choice and regulation of expression of CD133 in disease and development we have performed a comprehensive assessment of TSS activity using genome wide assay of transcription initiation.

Using transcriptional initiation events from a representative panel of 72 developmental, cancer, and normal CAGE (De Hoon and Hayashizaki, 2008; Hoskins et al., 2010; Kurosawa et al., 2010) libraries we have characterized PROM1 promoter utilization, confirming the activity of four out of five known promoters (P1–P4) and one novel alternate promoter (P6). P1 and P3 are consistently utilized in normal tissues, cancer, and development while P2 is active in developmental samples. A novel, proximal promoter P6 was identified in our high-resolution CAGE assays of a CD133^+^ subpopulation derived from melanoma cell lines and independently confirmed using 5′-RACE PCR. Initiation from P6 results in a significantly shorter 5′ untranslated region (UTR).

### Expression of P6

The discovery of P6 by genome wide CAGE assay is supported by existing *in silico* predictions and by comparison with exon array probes overlapping regions of PROM1 TSS. PROM1 transcripts initiate at P6 in cancer tissues, CD133^+^ melanoma cells, adult tissues, and stem cell enriched populations, including CD133^+^ selected cells from four primary Ewing sarcoma samples for which stem cell behavior has been established (Jiang et al., 2010).

### Transcriptional regulation of P6

Comparison of 149 core promoters of TSS found to be consistently co-expressed with the novel PROM1 P6 promoter in at least four out of five CD133^+^ melanoma cell lines identified motif enrichment for Sp1 binding sites present in all PROM1 promoters, in agreement with their importance in the CpG islands located around P1–3 (Gopisetty et al., 2012). A second enriched motif, HMGI/Y, was found in the P6 promoter and also the testis-specific promoter P4. HMG family proteins are ubiquitously expressed nuclear proteins which regulate transcription and chromatin structure (Reeves and Beckerbauer, 2001) and have role in differentiation, tumor progression, and malignancy (Wisniewski and Schwanbeck, 2000) by controlling genes involved in tumor initiation, invasion, cell proliferation, and angiogenesis (Reeves et al., 2001). HMGI/Y (HMG1A) is usually expressed at low levels in adult tissues, but found at high expression levels in embryonic and neoplastic tissues (Chiappetta et al., 1996), its aberrant expression has been associated with tumorigenesis (Tkachenko et al., 1997) and high expression is a requirement for the production of CXC ligand 1, a major effector of tumor growth(Amiri et al., 2006). Both isoforms (HMGI and HMGY) are expressed in neuroblastic tumors, with higher levels in less differentiated tumor (Giannini et al., 2000). In gliomas, HMGI/Y expression correlates with malignancy, proliferation, and invasion (Pang et al., 2011). High levels of HMGI/Y are found in more aggressive tumors and correlate with poor prognosis and are associated with a stem-like state (Shah and Resar, 2012). In addition, ENCODE ChIP-seq data indicates binding of two transcription factors (POUF2 and NF-kb) known to interact with HMGI/Y (Reeves et al., 2001) immediately downstream of P6 (POUF2) and in an upstream enhancer (NF-kb). Both Sp1 and HMGI/Y are expressed in CD133^+^ melanoma cell lines. We did not find them to be differentially expressed when compared to CD133-depleted cells, although HMGI/Y undergoes extensive post-translational modifications which influence its binding properties (Bianchi and Agresti, 2005).

### Epigenetic landscape

Given the role of HMGI/Y in modifying chromatin structure we explored epigenetic changes of the upstream PROM1 region in ENCODE cell line data. Based on ChIP-qPCR analysis, methylation is thought to affect CD133 expression only in cell lines but not in primary tissues (Pellacani et al., 2011), although methylation of P2 is thought to be tissue specific (Pleshkan et al., 2008). As expected, the CpG island close to P1–P3 was found to be differentially methylated between different ENCODE cell lines, with h-ESC and hepG2 being free of methylation and leukemic (K562) and lymphoblastoid (GM12878) cell lines showing methylation.

In addition, P1–3 were found to be polycomb-repressed in all surveyed cell line types with the exception of h1-ESC and, to a lesser extent, hepG2 which exhibit active promoters. Interestingly, h1-ESC showed signs of transcriptional transition between P1–3 and P6 which in combination with the active enhancer region upstream of P6 might explain increased transcriptional activity found in h1-ESC RNA-seq data around P6.

## Summary

By combining comprehensive bioinformatics analysis of genome-wide exon array and exhaustively and consistently sequenced CAGE samples across a broad range of cell and tissue types and a series of melanoma cell lines, it has been possible to reveal a strong association between a specific new promoter and clonogenic CD133^+^ cells. Together, these findings provide evidence of multiple regulatory events contributing to the diversity of PROM1 expression and indicate a potential role for HMGI/Y in combination with epigenetic changes to initiate transcription from P6 in less differentiated cells or stem cells, resulting in an upregulation of CD133. This study provides one of the few links between expression of a stem cell marker and its likely regulation.

## Author contributions

Ramakrishna Sompallae: Collection and/or assembly of data, Data analysis and interpretation, Manuscript writing; Oliver Hofmann: Collection and/or assembly of data, Data analysis and interpretation, Manuscript writing; Craig Gedye: Conception and design, Provision of study material or patients, Manuscript writing; Andreas Behren: Study design and conception, provision of study material or patients, Manuscript writing; Morana Vitezic: Data analysis and interpretation; Sylvie Devalle: Collection and/or assembly of data; Otavia L. Caballero: Collection and/or assembly of data, Elizabeth R. Lawlor: Provision of study material or patients, Collection and/or assembly of data, Manuscript writing, Final approval of manuscript; Jonathan Cebon: Provision of study material, Conception and design, Financial support, Review of manuscript; Winston Hide: Conception and design, Financial support, Data analysis and interpretation, Manuscript writing, Final approval of manuscript.

### Conflict of interest statement

The authors declare that the research was conducted in the absence of any commercial or financial relationships that could be construed as a potential conflict of interest.

## Supplementary material

Figure S1 **PROM1 promoter activity using exon arrays in additional tissue panels**. Transcript wide expression pattern of PROM1 measured by Affymetrix exon arrays with specific probes targeting exonic regions. **Left panel**: colon, pancreas, testis, and kidney expressing P6. **Right panel**: spleen, prostate, muscle, and thyroid expressing P1-P2.

Table S1 **Tissues surveyed for PROM1 promoter activity**.

Table S2 **Melanoma cell cultures used in the study and the patient disease stage at collection**.

Table S3 **Exon arrays used in the study**.

Table S4 **CD133hi_set_4_5**.
