# Supplementary material for: A comprehensive promoter landscape identifies a novel promoter for CD133 in restricted tissues, cancers, and stem cells
Source: Front Genet. 2013 Oct 29;4:209. doi: 10.3389/fgene.2013.00209 (PMC3810939; doi:10.3389/fgene.2013.00209)
Supplement: Figure S1 — PROM1 promoter activity using exon arrays in additional tissue panels. Transcript wide expression pattern of PROM1 measured by Affymetrix exon arrays with specific probes targeting exonic regions. Left panel: colon, pancreas, testis, and kidney expressing P6. Right panel: spleen, prostate, muscle, and thyroid expressing P1-P2. [file DataSheet1.ZIP › 62018_Hofmann_Supplementary Table S1.DOCX]

Supplementary Table S1. Melanoma cell cultures used in the study and the patient disease stage when collected.

| Cell line | Sex | Age at Primary diagnosis | Age at sample collection | Stage^a^ | Site of Tissue | Status |
| --- | --- | --- | --- | --- | --- | --- |
| LM-Mel-14 | F | 44 | 45 | IIIB | Right inguinal lymph node | dead |
| LM-Mel-34 | F | 74 | 75 | IIIC | Left axillary lymph node | alive |
| LM-Mel-42 | M | 65 | 67 | IV | Splenic metastasis | dead |
| LM-Mel-47 | F | 54 | 56 | IIIC | Left inguinal lymph node | dead |
| LM-Mel-62 | M | 60 | 72 | IIIC | Right auxillary lymph node | alive |

a. disease stage at the time of tissue collection
