# Supplementary material for: A comprehensive promoter landscape identifies a novel promoter for CD133 in restricted tissues, cancers, and stem cells
Source: Front Genet. 2013 Oct 29;4:209. doi: 10.3389/fgene.2013.00209 (PMC3810939; doi:10.3389/fgene.2013.00209)
Supplement: Figure S1 — PROM1 promoter activity using exon arrays in additional tissue panels. Transcript wide expression pattern of PROM1 measured by Affymetrix exon arrays with specific probes targeting exonic regions. Left panel: colon, pancreas, testis, and kidney expressing P6. Right panel: spleen, prostate, muscle, and thyroid expressing P1-P2. [file DataSheet1.ZIP › 62018_Hofmann_Supplementary Table S2.DOCX]

Supplementary Table S2. Exon arrays used in the study

| Tissue type | CD133 expression | Number of arrays |
| --- | --- | --- |
| Mesenchymal stem cell | low | 3 |
| Neural crest stem cell | high | 3 |
| differentiated NCSC | low | 1 |
| Ewing sarcoma | high | 7 |
| Ewing sarcoma | low | 3 |
| Breast | Not done | 3 |
| Cerebellum | Not done | 3 |
| Colon | Not done | 10 |
| Heart | Not done | 3 |
| Kidney | Not done | 3 |
| Liver | Not done | 3 |
| Muscle | Not done | 3 |
| Pancreas | Not done | 3 |
| Prostate | Not done | 3 |
| Spleen | Not done | 3 |
| Testis | Not done | 3 |
| Thyroid | Not done | 3 |
