# Supplementary figures and images for: A comprehensive promoter landscape identifies a novel promoter for CD133 in restricted tissues, cancers, and stem cells
Source: Front Genet. 2013 Oct 29;4:209. doi: 10.3389/fgene.2013.00209 (PMC3810939; doi:10.3389/fgene.2013.00209)

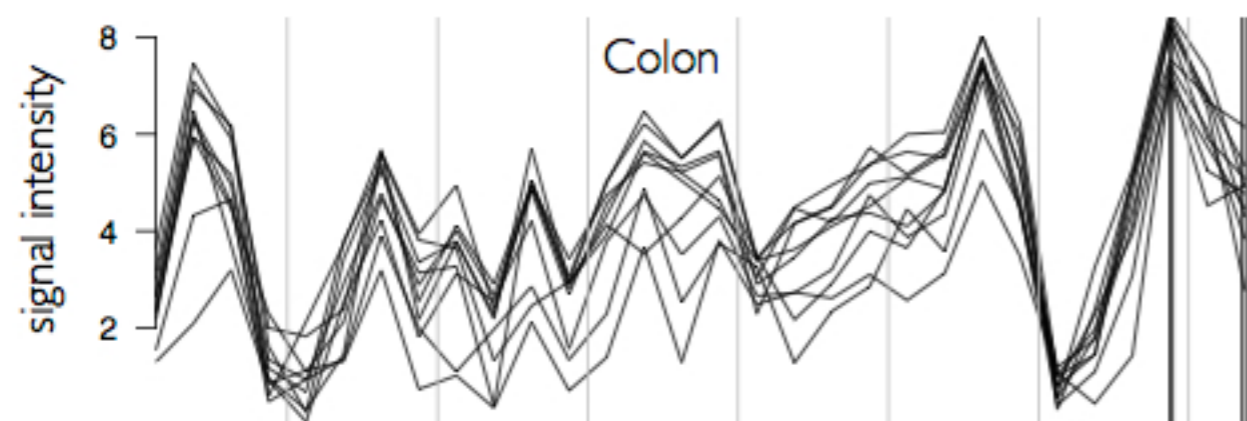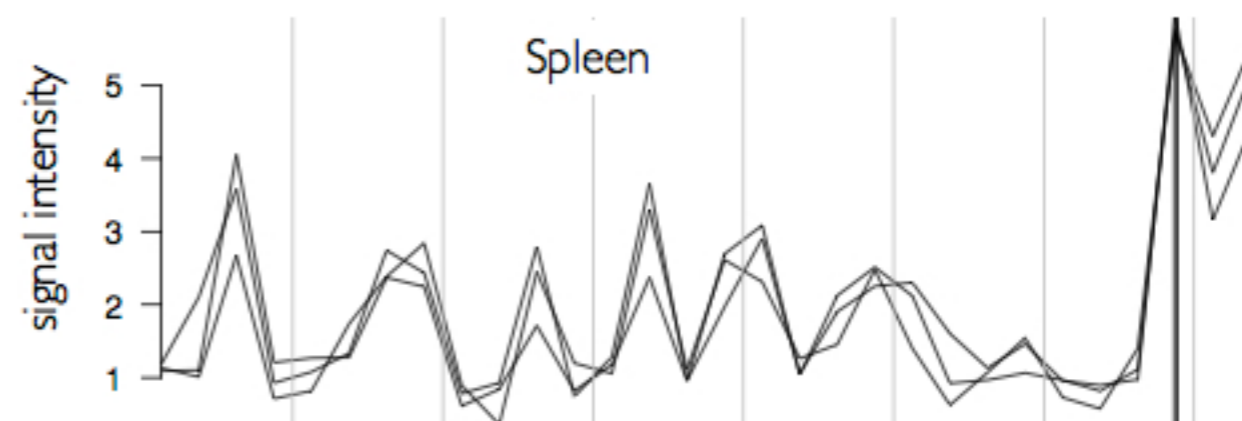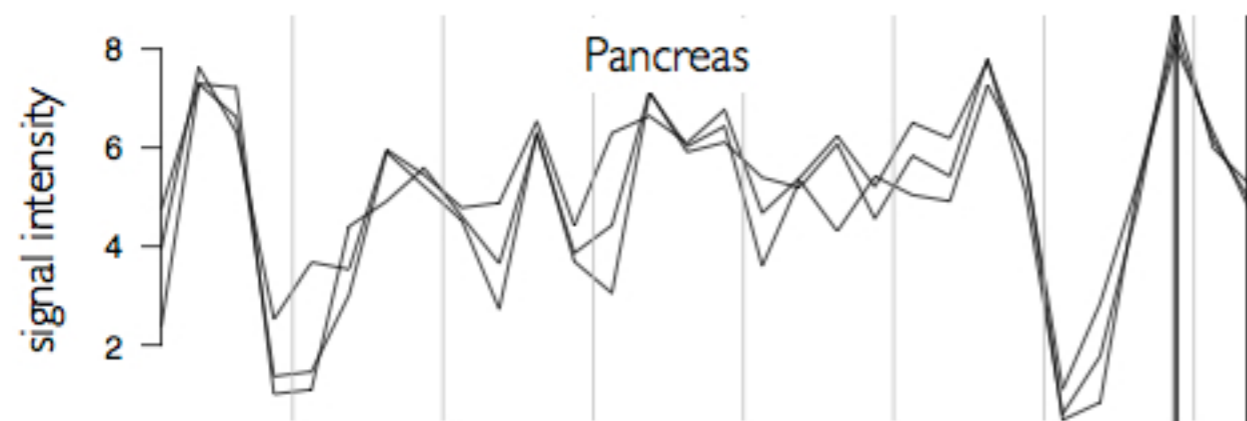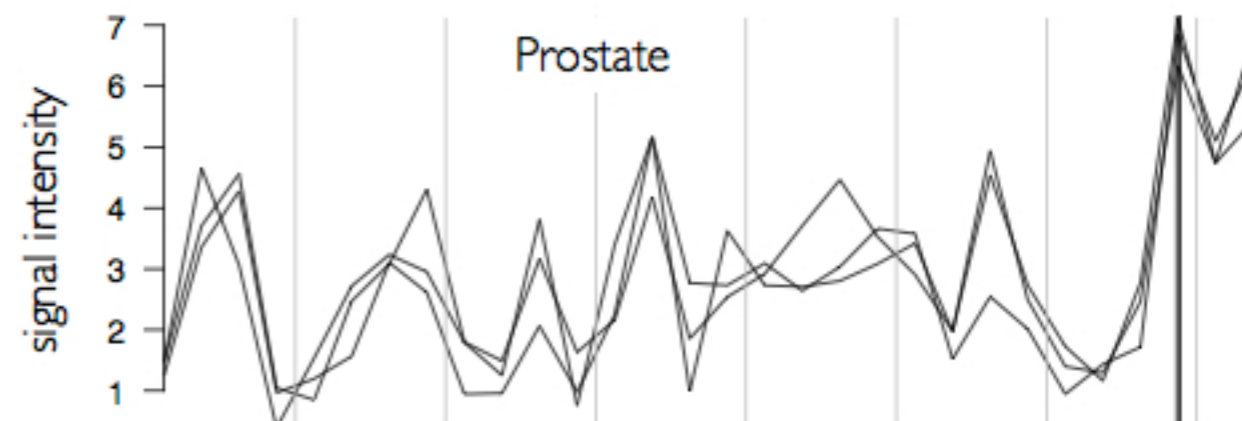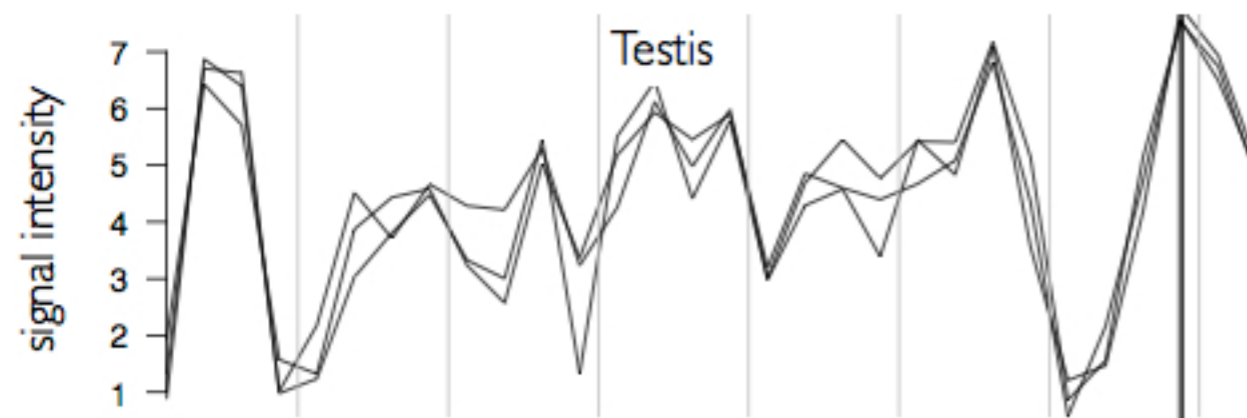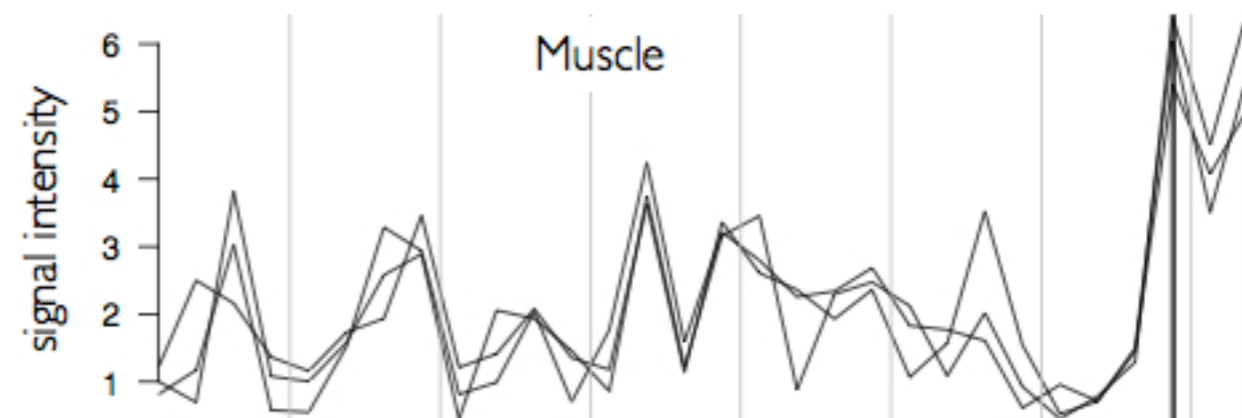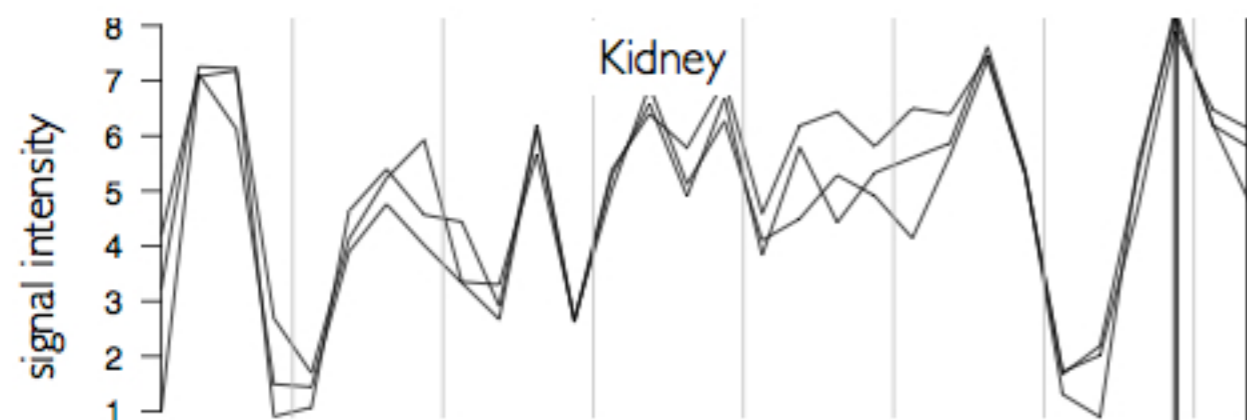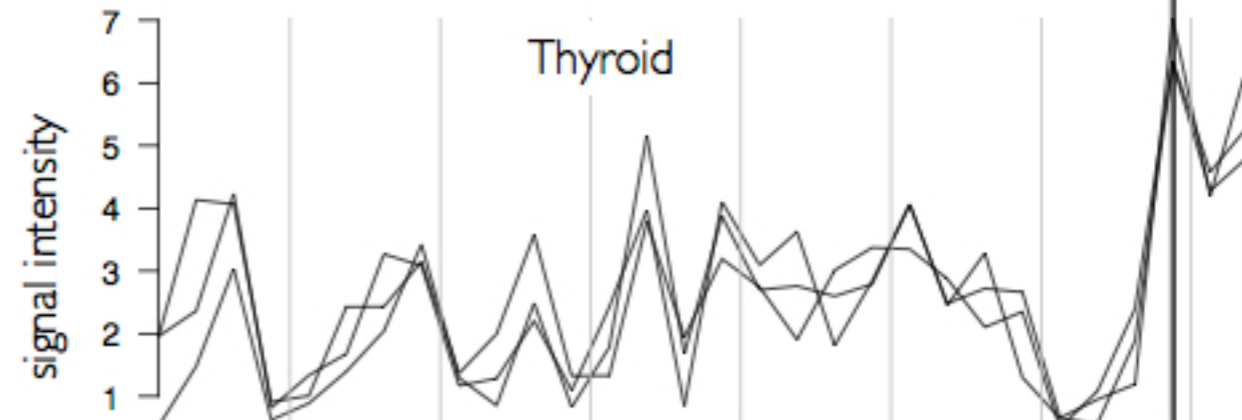

Supplement: Figure S1 — PROM1 promoter activity using exon arrays in additional tissue panels. Transcript wide expression pattern of PROM1 measured by Affymetrix exon arrays with specific probes targeting exonic regions. Left panel: colon, pancreas, testis, and kidney expressing P6. Right panel: spleen, prostate, muscle, and thyroid expressing P1-P2. [file DataSheet1.ZIP › 62018_Hofmann_Supplementary Figure S1.PDF]
