# Supplementary material for: A comprehensive promoter landscape identifies a novel promoter for CD133 in restricted tissues, cancers, and stem cells
Source: Front Genet. 2013 Oct 29;4:209. doi: 10.3389/fgene.2013.00209 (PMC3810939; doi:10.3389/fgene.2013.00209)
Supplement: Figure S1 — PROM1 promoter activity using exon arrays in additional tissue panels. Transcript wide expression pattern of PROM1 measured by Affymetrix exon arrays with specific probes targeting exonic regions. Left panel: colon, pancreas, testis, and kidney expressing P6. Right panel: spleen, prostate, muscle, and thyroid expressing P1-P2. [file DataSheet1.ZIP › 62018_Hofmann_Supplementary Table S3.DOC]

| Sample ID | Tissue | Class |
| --- | --- | --- |
| LM-Mel-14pos | melanoma | CD133^hi^ |
| LM-Mel-34pos | melanoma | CD133^hi^ |
| LM-Mel-42pos | melanoma | CD133^hi^ |
| LM-Mel-47pos | melanoma | CD133^hi^ |
| LM-Mel-62pos | melanoma | CD133^hi^ |
| LM-Mel-14neg | melanoma | CD133^lo^ |
| LM-Mel-34neg | melanoma | CD133^lo^ |
| LM-Mel-42neg | melanoma | CD133^lo^ |
| LM-Mel-47neg | melanoma | CD133^lo^ |
| LM-Mel-62neg | melanoma | CD133^lo^ |
| HAH | cecum | Normal |
| HAI | kidney | Normal |
| HAQ | large intestine | Normal |
| HAS | large intestine | Normal |
| HAL | liver | Normal |
| HBP | liver | Normal |
| HBR | pancreas | Normal |
| HBB | prostate | Normal |
| HAF | rectum | Normal |
| HBO | rectum | Normal |
| HAK | stomach | Normal |
| HCQ | testis | Normal |
| HCY | testis | Normal |
| HER | mammary gland | Cancer |
| HCD | colon | Cancer |
| HCE | colon | Cancer |
| HCF | lung | Cancer |
| HCG | lung | Cancer |
| HEU | prostate | Cancer |
| HEV | prostate | Cancer |
| HEW | prostate | Cancer |
| HBY | liver | Cancer |
| HEN | liver | Cancer |
| HEQ | liver | Cancer |
| HAG | cecum | Cancer |
| HAJ | kidney | Cancer |
| HAR | large intestine | Cancer |
| HAT | large intestine | Cancer |
| HBQ | liver | Cancer |
| HBS | pancreas | Cancer |
| HAE | rectum | Cancer |
| HCH | skin | Cancer |
| HCB | adrenal gland | Cancer |
| HEM | bone marrow | Cancer |
| HEP | bone marrow | Cancer |
| HAA | monoblast | Cancer |
| HAB | monocyte | Cancer |
| HAC | monocyte | Cancer |
| HCI | brain | Developmental |
| HCJ | brain | Developmental |
| HDL | brain | Developmental |
| HDM | brain | Developmental |
| HDN | brain | Developmental |
| HDO | brain | Developmental |
| HDP | brain | Developmental |
| HDQ | brain | Developmental |
| HCK | colon | Developmental |
| HCL | colon | Developmental |
| HDI | heart | Developmental |
| HDJ | heart | Developmental |
| HDK | heart | Developmental |
| HDX | kidney | Developmental |
| HDY | kidney | Developmental |
| HDZ | kidney | Developmental |
| HDU | liver | Developmental |
| HDV | liver | Developmental |
| HDW | liver | Developmental |
| HCA | lung | Developmental |
| HDR | lung | Developmental |
| HDS | lung | Developmental |
| HDT | lung | Developmental |
| HEA | muscle | Developmental |
| HEB | muscle | Developmental |
| HEC | muscle | Developmental |
| HCM | spleen | Developmental |
| HCN | thymus | Developmental |
| HDC | whole body | Developmental |
| HDD | whole body | Developmental |
| HDE | whole body | Developmental |
| HDF | whole body | Developmental |
| HDG | whole body | Developmental |
| HDH | whole body | Developmental |
